# How Maternal Nutritional and Mental Health Affects Child Health During Pregnancy: A Narrative Review

**DOI:** 10.7759/cureus.48763

**Published:** 2023-11-13

**Authors:** Afsana Naaz, Komal N Muneshwar

**Affiliations:** 1 Medicine, Jawaharlal Nehru Medical College, Datta Meghe Institute of Higher Education and Research, Wardha, IND; 2 Community Medicine, Jawaharlal Nehru Medical College, Datta Meghe Institute of Higher Education and Research, Wardha, IND

**Keywords:** child health, development, child, maternal nutrition, pregnancy, mental health

## Abstract

Throughout pregnancy, the mother's nutritional and mental health significantly influences the kid's long-term growth and wellness. This review's objective is to provide a comprehensive assessment of the literature on the link between pregnancy nutrition and a sound mind and a foetus' growth, considering factors in the physical, cognitive, emotional, and social domains. A balanced, nutrient-rich diet is crucial for the baby to grow and develop properly during pregnancy. An appropriate diet of significant macronutrients and micronutrients supports good foetal organ development, cognitive function, and immune system resiliency. For instance, studies have linked iron and omega-3 fatty acids to a reduced risk of developmental delays and improved cognitive performance. Contrarily, malnutrition in mothers, such as undernutrition or excessive weight gain, has been connected with negative results, including low birth weight, poor neurodevelopment, and increased susceptibility to chronic diseases in later life. The mother's mental health, including emotional equilibrium and psychological stability, significantly impacts the child's development. Stress, anxiety, and depression experienced by mothers during pregnancy harm the developing foetus and increase the risk of cognitive, behavioural, and emotional difficulties in the offspring. The growing foetus is exposed to high levels of stress hormones due to chronic maternal stress, which might alter the fetus's brain's shape and function. Factors influencing child development outcomes include maternal-infant attachment, breastfeeding start and duration, and general caring practices. A comprehensive approach is critical since new research indicates a synergistic relationship between maternal nutrition and mental health. Inadequate nutritional intake might result from maternal mental health problems that interfere with appetite control and eating habits. On the other hand, inadequate maternal nutrition may raise maternal stress and result in mental health problems. Therefore, integrative therapies focusing on both areas are essential to maximise child development results. In conclusion, maternal nutrition and mental health during pregnancy significantly impact a child's development in various domains. Understanding the complex relationships between maternal nutrition and mental health is necessary to develop effective therapies and promote the most remarkable results for children. Further research is needed to understand better the underlying mechanisms to develop evidence-based recommendations for optimal mother care throughout pregnancy. The need of this review is to know how maternal health, physical or mental affects the child's development and how we can further prevent it by taking precautions during pregnancy.

## Introduction and background

The journey from conception to birth encompasses a critical period of development that significantly shapes a child's future well-being. During this time, many factors come into play, influencing the growth and development of the unborn child. Among these factors, maternal nutrition and sound mind are vital in determining a child's development trajectory. Understanding the interplay between a mother's nutritional status and mental well-being during pregnancy is crucial for ensuring optimal outcomes for both the mother and child [1]. This is long recognised as a significant factor influencing foetal growth and development and maternal nutritional condition throughout pregnancy. Maternal nutrition is a crucial component of prenatal care since the growing foetus ultimately depends on its mother for critical nutrients. An adequate intake of essential nutrients such as proteins, carbs, fats, vitamins, and minerals is vital to promote foetal organ development, skeletal growth, and normal physiological functioning. Developmental delays, restriction perinatal outcomes, and reduced newborn weight are just a few of the adverse effects of inadequate consumption of these critical nutrients [2]. Additionally, even after the pregnancy, maternal nutritional inadequacies may affect the child's growth and health [3]. Inborn chronic illnesses, including obesity, diabetes, cardiovascular disease, and poor cognitive development, have been linked in studies to mother malnutrition during pregnancy. These findings stress the relevance of sufficient mother nutrition throughout pregnancy and maternal nutrition's crucial role in determining the child's long-term health trajectory. Maternal mental health and diet substantially influence the development of the foetus and the offspring.

Pregnant women may encounter numerous state-of-mind problems, such as stress, throughout pregnancy, which can increase emotional sensitivity. Disorders of the mother's mental health during pregnancy harm the baby's development regarding emotion, cognition, and behaviour [4]. Conditions of the maternal mind can interfere with the physiological and biochemical harmony of the mother's body, affecting foetal development. Cortisol, a hormone high during maternal stress or worry, can pass through the placental barrier and reach the growing foetus [5]. Long-term exposure to high amounts of stress hormones can cause abnormal foetal brain development, impairing regions in charge of emotional control, attention, and memory. As a result, children born to moms with untreated mental health issues during pregnancy may be more likely to grow up with behavioural problems, cognitive issues, and psychiatric illnesses [6]. Additionally, the parent-child bond and maternal mental health might indirectly impact children's growth [7]. For instance, maternal depression may hinder a mother's capacity to give sensitive and responsive care to her kid, disrupting early attachment and socio-emotional growth. According to research, infants exposed to mother depression during the antenatal and postnatal period may have more excellent rates of insecure attachment, emotional problems, and poor social skills [8]. 

Maternal diet and mental health are crucial during pregnancy, and treatments designed to promote the best results are receiving more and more attention [9]. Nutritional counselling is frequently included in prenatal care programmes, focusing on a well-balanced diet to guarantee optimal nutrient intake for both the mother and foetus. To effectively detect and manage maternal mental health difficulties, mental health screening and support services are being incorporated into routine prenatal care [10]. Maintaining a healthy weight throughout pregnancy is essential for the well-being of the mother and the unborn child. The chance of gestational diabetes, hypertension, and difficulties after labour can all be raised by excessive weight gain. By increasing the risk of obesity and accompanying conditions such as cardiovascular disease and diabetes, it may also improve the child's long-term health. In contrast, poor weight growth might cause low birth weight and developmental issues [11,12]. Doing a review on this topic is vital for the well-being of individuals, communities, and societies, as it leads to improved health outcomes, more effective policies and practices, and better opportunities for the future.

## Review

Methodology 

In this review, we look at the following: how the growth of the unborn kid is impacted by the mother's nutrition and mental health. The electronic databases PubMed, Medline, Embase, and Google Scholar were used to search the English-language literature. Keywords used include "pregnancy" and "child development " (((Pregnancy Title/Abstract]) OR (Nutrition[Title/Abstract])) OR (mental health[Title/Abstract])) OR ("pregnancy" [MeSH Terms]) AND (("child health" [Title/Abstract]) OR (Child health [Title/Abstract])) OR ("child development"[MeSH Terms]). The authors' own knowledge of the subject and experience in the area provided support for the archiving of pertinent publications. Research in English, older research, and studies solely focused on the significance of a healthy mother having a healthy kid were all the criteria of the articles that were included in this study.

This review article is on how maternal nutritional and mental health affects the child's health during pregnancy. The mother's health is connected with the child's health. What she is eating and any nutritional deficiency will show their effects on the development and growth of the child. Any type of addiction or consumption of alcohol will also cause an ill effect on the child's health; in the same way, maternal mental health also has a huge importance on the child. If the mother is suffering from any mental disorder during her pregnancy, it can also have effects on the child's health.

Impact of nutritional health during pregnancy on foetal development 

It is crucial to have a healthy diet while expecting a child. The developing fetus relies entirely on the mother's diet for essential nutrients, vitamins, and minerals vital for development. A balanced and nutrient-rich diet ensures the optimal growth of the baby's organs, brain, and immune system. A deficiency in essential nutrients, such as folic acid, iron, calcium, and omega-3 fatty acids, can lead to adverse outcomes for the child. During pregnancy, consuming a balanced diet with macronutrients, micronutrients, and vitamins is necessary. Macronutrients, such as carbohydrates, proteins, and fats, provide energy and support fetal growth and development. Micronutrients, such as iron, calcium, and folate, are essential for red blood cell formation, bone growth, and preventing congenital disabilities. Vitamin D aids calcium absorption, while vitamin C promotes tissue repair and boosts the immune system. Other essential vitamins for pregnant women include vitamin A for vision and cell growth, vitamin E for cell protection, and B-complex vitamins for metabolism and brain development. It is advisable to consult a healthcare professional for personalised nutrient recommendations during pregnancy. The recommended dietary allowances for non-pregnant women and estimated average requirements and recommended dietary allowances for pregnant women have been highlighted in Table 1.

**Table 1:** Recommended dietary allowances for non-pregnant women and estimated average requirements and recommended dietary allowances for pregnant women. Source: Ref. [13]

Nutrient	Recommended dietary allowances, adult non-pregnant women	Estimated average requirements, pregnant women	Recommended dietary allowances, pregnant women
Retinol (μg/day)	700	550	770
Calciferol (μg/day)	15	10	15
Vitamin B12 (mg/day)	2.4	2.2	2.6
Ascorbic acid(mg/day)	75	70	85
Calcium (mg/day)	1000	800	1000
Iodine (μg/day)	150	160	220
Iron (mg/day)	18	22	27

Folic Acid

Folic acid is crucial for proper fetal development, especially in the early stages. It plays a vital role in deoxyribose nucleic acid synthesis, cell division, and tissue formation. A growing fetus is likelier to have neural tube abnormalities if not given enough folic acid throughout pregnancy. Severe brain and spinal cord abnormalities, known as neural tube defects, include spina bifida, anencephaly, and encephalocele. These conditions can lead to lifelong disabilities or even be fatal. Besides neural tube defects, folic acid deficiency may impact other organs. Folic acid is crucial for synthesising red blood cells, and inadequate levels can cause megaloblastic anaemia in both the mother and the fetus. Anaemia reduces oxygen-carrying capacity, affecting the development of organs that require sufficient oxygen, such as the brain and heart [14]. Furthermore, folic acid deficiency may interfere with the normal development of the heart and cardiovascular system, increasing the child's risk of congenital heart defects. It can also affect the gastrointestinal tract, kidney, and other organs, as folic acid is involved in their proper formation and functioning. To mitigate these risks, pregnant women must ensure adequate folic acid intake. The recommended daily folic acid information during pregnancy is typically higher than for non-pregnant women. Prenatal supplements containing folic acid are commonly prescribed to meet the increased requirements. Additionally, consuming nutrients ample in folate, such as green vegetables, legumes, citrus fruits, and fortified grains, can help prevent folic acid deficiency and support healthy organ development in the child [15].

Iron

A mother's iron status during pregnancy can significantly influence the baby's organ development. The formation of haemoglobin, the protein in red blood cells that transports oxygen throughout the body, requires iron, a vital element. During pregnancy, the demand for iron increases to support the growing fetus and placenta. Insufficient iron intake can lead to iron deficiency anaemia in the mother, reducing oxygen-carrying capacity. Inadequate iron levels can impact the developing organs of the child. The brain requires a sufficient oxygen supply for proper growth and development. Iron deficiency can impair oxygen delivery to the brain, potentially leading to cognitive and behavioural problems in the child, including decreased Intelligence quotient, poor attention span, and learning difficulties [16]. Iron deficiency during pregnancy can also affect the cardiovascular system of the child. Iron is necessary to form red blood cells and synthesise various enzymes involved in energy production and cardiac function. Insufficient iron can lead to reduced blood volume and compromised cardiac output in the fetus, potentially resulting in developmental issues in the heart and other organs. Furthermore, iron plays a crucial role in the immune system, and deficiency during pregnancy can weaken the child's immune response, making them more susceptible to infections and diseases [17]. Pregnant women must get enough iron to avoid these potential effects on organ development. Lean meats, chicken, fish, legumes, fortified cereals, and dark leafy greens are foods high in iron that help you reach your goals. Prenatal vitamins containing iron are frequently advised to satisfy the increased iron needs during pregnancy. Effective treatment and management of iron shortage during pregnancy depend on regular monitoring of iron levels and consultation with medical specialists [18].

Vitamin D

Prenatal vitamin D insufficiency can significantly impact the baby's health and development. Due to its assistance in the absorption of calcium and phosphorus, vitamin D is essential for the development of bones and teeth. Vitamin D deficiency in the mother can prevent the baby's bones from properly mineralising, resulting in skeletal abnormalities, such as rickets, a disorder marked by weak and fragile bones [19]. Beyond bone health, vitamin D is also involved in regulating the immune system. Maternal deficiency can impair the development and function of the child's immune system, increasing the risk of infections and allergies during infancy and childhood. Furthermore, emerging research suggests that maternal vitamin D deficiency may be linked to an increased risk of specific long-term health issues in the child. Studies have found associations between low maternal vitamin D levels during pregnancy and an elevated risk of childhood asthma, wheezing, and respiratory tract infections. Evidence suggests a potential connection between maternal vitamin D deficiency and developmental delays in children [20]. To mitigate these risks, pregnant women must maintain adequate vitamin D levels. This can be achieved through sunlight exposure, dietary sources (such as fatty fish, fortified dairy products, and eggs), and vitamin D supplements, as advised by healthcare professionals. Ensuring sufficient vitamin D intake during pregnancy is essential for promoting optimal bone development, a robust immune system, and overall healthy growth and development in the child [21].

Vitamin B12

During pregnancy, when the neural tube and brain of the foetus are developing, vitamin B12 is especially significant since it is essential for the development and proper operation of the nervous system. A deficiency of this vitamin in pregnant women's diet can significantly affect the child's development. Insufficient intake of vitamin B12 during pregnancy can lead to a condition known as maternal vitamin B12 deficiency, which can result in various adverse effects on the developing fetus. It has been associated with an increased risk of neural tube defects, such as spina bifida, and impaired brain development, potentially leading to cognitive and neurological impairments in the child [22]. Moreover, vitamin B12 deficiency in pregnancy has been linked to an increased risk of preterm birth, low birth weight, and developmental delays in the child. The deficiency can affect the myelination process of nerve fibres, which is crucial for proper nerve signal transmission and coordination. To prevent these complications, pregnant women need to ensure an adequate intake of vitamin B12 through a balanced diet or supplements. Foods rich in vitamin B12 include meat, fish, dairy products, and fortified cereals. Regular prenatal check-ups and consultations with healthcare professionals can help identify and address any nutritional deficiencies, ensuring the healthy development of the fetus and reducing the risk of long-term complications [23].

Iodine

Insufficient iodine intake can impair thyroid hormone production, crucial for brain development. This deficiency can result in cognitive and neurological impairments, including lower Intelligence quotient, learning difficulties, and memory and attention problems. Physical growth may also be affected, leading to stunted stature and impaired motor skills. Iodine deficiency increases the risk of hearing impairments speech and language disorders and weakens the immune system, making children more vulnerable to infections [24]. To prevent these consequences, pregnant women should consume an adequate amount of iodine, approximately 220-250 micrograms daily. Food sources such as iodised salt, seafood, seaweed, dairy products, and eggs are recommended. In some cases, iodine supplements may be necessary. Maintaining a balanced and nutritious diet during pregnancy is essential for the child's healthy growth. Consultation with a healthcare professional is advised for concerns regarding iodine intake during pregnancy [25].

Impact of addiction during pregnancy on foetal development 

Pregnancy-related addiction can have significant, long-lasting effects on a child's development. Abuse of any substance, including alcohol, cigarettes, or illegal narcotics, can harm the developing foetus in several ways.

Physical and neurological effects: Substance abuse during pregnancy can lead to physical abnormalities and developmental issues in the child. For instance, alcohol consumption by the mother can cause fetal alcohol syndrome, resulting in facial deformities, growth deficiencies, and intellectual disabilities. Illicit drugs can also disrupt normal brain development, leading to cognitive impairments and behavioural problems.

Preterm birth and poor infant weight: Substance abuse increases the possibility of premature birth (before 37 weeks) and delivering a baby with low birth weight. These factors can cause a range of health issues for the child, along with respiratory difficulties, feeding issues, and developmental delays [26].

Neonatal abstinence syndrome: If a pregnant woman is addicted to opioids, her baby may have neonatal abstinence syndrome [27].

Behavioural and cognitive problems: Children exposed to substances during pregnancy may be at a higher risk of behavioural issues, including hyperactivity, impulsivity, and learning difficulties. They may struggle with attention span, memory, and academic performance throughout childhood and adolescence. It is crucial for pregnant women struggling with addiction to seek appropriate medical care and support. Treatment programs, including substance abuse counselling, detoxification, and rehabilitation, can help reduce the risks and provide a healthier environment for the developing child. Healthcare professionals can offer guidance and support to minimise the impact of addiction on child development. Early intervention and support services for the mother and child can also help mitigate potential long-term problems [28].

Impact of a mother's mental health on foetal development

Maternal mental conditions during pregnancy can significantly impact child development. Positive maternal mental health promotes bonding, secure attachment, and healthy social-emotional development. Maternal stress, anxiety, and depression can increase cortisol levels, potentially affecting the child's brain development and stress response. A greater probability of emotional, psychological, and cognitive disorders in the unborn infant is related to concerns with the mother's mental health. Prioritising maternal mental well-being through support, treatment, and a nurturing environment is crucial for promoting optimal child development. Healthcare providers and support networks are vital in identifying and addressing maternal mental health concerns while pregnant. Prevalence estimates for certain mental illnesses during pregnancy have been highlighted in Table 2.

**Table 2:** Prevalence estimates for certain mental illnesses during pregnancy. Source: Ref. [29]

Disease	Disability in the child	Estimated prevalence (%)
Depressive disorders	Major depression	15-23
Anxiety disorders	General anxiety disorder	9.5
	Panic disorder	1-3
	Post-traumatic stress disorder	5.6
	Obsessive-compulsive disorder	0.4-1.4
Eating disorders	Anorexia only	1.6
	Bulimia only	1.8
	Both anorexia and bulimia	0.9

Depression

Maternal depression can have essential impacts on the foetus. Children of depressive moms are more likely to experience behavioural and emotional issues, cognitive delays, and social and emotional difficulties. They may struggle with forming secure attachments, have impaired social interactions, and face an increased risk of developing mental health issues themselves. Early identification and intervention are crucial to minimise the adverse effects. Treatment for maternal depression and supportive interventions focusing on enhancing the parent-child relationship can help mitigate the impact on child development and promote a healthier outcome [30].

*Anxiety* 

Maternal anxiety can have significant impacts on child growth. Babies of anxious mothers can experience anxiety, exhibiting excessive worrying, fearfulness, and avoidance. Maternal stress can also affect the parent-child relationship, leading to difficulties with bonding and attachment. Additionally, children may exhibit cognitive and academic challenges, including impaired attention and problem-solving skills. Early identification and intervention are essential for addressing maternal anxiety and supporting the mother and child. Treatment options, such as therapy and stress reduction techniques, can help alleviate maternal anxiety and mitigate its impact on child development [31].

Stress

Maternal stress can have significant impacts on child development. Prolonged or high maternal stress levels can disrupt the prenatal environment, potentially affecting the child's brain development, emotional regulation, and stress response. Children of stressed mothers may be at increased risk of emotional and behavioural difficulties, cognitive delays, and impaired social interactions. Maternal stress can also influence the parent-child relationship and bonding. Early identification and support for maternal stress are essential for minimising its harmful effects. Stress management techniques, social support, and interventions that promote a nurturing environment can help mitigate the impact of maternal stress on child growth [32].

Discussion

Child health during pregnancy hinges on maternal nutrition and mental health. Nutrition is vital for optimal growth and development of the fetus, but lack of proper nutrition leads to different complications that affect the general health of this person throughout his lifetime. However, maternal mental health is important since it determines prenatal outcomes. High degrees of stress, anxiety, and depression may result in premature delivery, low birth weight, and learning disabilities due to defects in brain development. Poor mental health can also result in poor dietary choices that can further deteriorate health status. It calls for a holistic approach aiming at supporting mothers and promoting healthy pregnancy.

Limitation

The limitation of this review is that culture and customs and habits related to food and nutrition during pregnancy are not covered, as well as some superstitions related to food habits during pregnancy, which affects maternal health and child health.

## Conclusions

Additionally, it can raise the child's chance of developing long-term health issues, including diabetes, obesity, and cardiovascular disease. On the other hand, a mother's mental health tremendously affects the prenatal environment and substantially impacts the kid's well-being. Several negative consequences have been connected to maternal stress, anxiety, and depression during pregnancy. High amounts of stress hormones, such as cortisol, can penetrate the placenta and impact the developing foetus, perhaps impairing brain development, causing emotional dysregulation, and altering stress responses in the kid. A higher risk of emotional and behavioural issues, delays in cognitive development, and even long-term mental health illnesses in children are linked to maternal anxiety and depression. In conclusion, maternal nutritional and mental health profoundly affect child health during pregnancy. Adequate nutrition is crucial for providing the necessary building blocks for the developing fetus and promoting optimal growth and development. A healthy, balanced diet high in essential nutrients is vital to lower the risk of problems and support the child's long-term health.

Education and counselling on proper nutrition and strategies to manage stress and promote mental well-being should be integral to prenatal care. Collaborative efforts between healthcare workers, specialists, and nutritionists are essential to ensure complete supervision for pregnant females and support healthy child development. We can improve future generations' overall health outcomes and well-being by addressing and optimising maternal nutritional and mental health. We can enhance future generations' general health and well-being by managing and improving mother's nutrition.

## References

[1] Marsál K (2002). Intrauterine growth restriction. Curr Opin Obstet Gynecol.

[2] May PA, Hasken JM, Baete A (2020). Fetal alcohol spectrum disorders in a Midwestern city: child characteristics, maternal risk traits, and prevalence. Alcohol Clin Exp Res.

[3] Chambers CD, Coles C, Kable J (2019). Fetal alcohol spectrum disorders in a Pacific Southwest City: maternal and child characteristics. Alcohol Clin Exp Res.

[4] Yajnik C (2006). Nutritional control of fetal growth. Nutr Rev.

[5] Barker DJ, Clark PM (1997). Fetal undernutrition and disease in later life. Rev Reprod.

[6] Gluckman PD, Pinal CS (2003). Regulation of fetal growth by the somatotrophic axis. J Nutr.

[7] Scholl TO (2011). Maternal iron status: relation to fetal growth, length of gestation, and iron endowment of the neonate. Nutr Rev.

[8] May PA, Hamrick KJ, Corbin KD (2014). Dietary intake, nutrition, and fetal alcohol spectrum disorders in the Western Cape Province of South Africa. Reprod Toxicol.

[9] Nguyen TT, Risbud RD, Chambers CD, Thomas JD (2016). Dietary nutrient intake in school-aged children with heavy prenatal alcohol exposure. Alcohol Clin Exp Res.

[10] van Bussel JC, Spitz B, Demyttenaere K (2006). Women's mental health before, during, and after pregnancy: a population-based controlled cohort study. Birth.

[11] Gausia K, Fisher C, Ali M, Oosthuizen J (2009). Antenatal depression and suicidal ideation among rural Bangladeshi women: a community-based study. Arch Womens Ment Health.

[12] Ringholm L, Nørgaard SK, Rytter A, Damm P, Mathiesen ER (2022). Dietary advice to support glycaemic control and weight management in women with type 1 diabetes during pregnancy and breastfeeding. Nutrients.

[13] Jouanne M, Oddoux S, Noël A, Voisin-Chiret AS (2021). Nutrient requirements during pregnancy and lactation. Nutrients.

[14] Molloy AM, Kirke PN, Brody LC, Scott JM, Mills JL (2008). Effects of folate and vitamin B12 deficiencies during pregnancy on fetal, infant, and child development. Food Nutr Bull.

[15] McPartlin J, Halligan A, Scott JM, Darling M, Weir DG. (1993). Accelerated folate breakdown in pregnancy. Lancet.

[16] Casanueva E, Pfeffer F, Drijanski A, Fernández-Gaxiola AC, Gutiérrez-Valenzuela V, Rothenberg SJ (2003). Iron and folate status before pregnancy and anemia during pregnancy. Ann Nutr Metab.

[17] Raut AK, Hiwale KM (2022). Iron deficiency anemia in pregnancy. Cureus.

[18] Merz LE, Achebe MO (2023). Iron deficiency in pregnancy: a health inequity. Am J Clin Nutr.

[19] Zhang H, Wang S, Tuo L, Zhai Q, Cui J, Chen D, Xu D (2022). Relationship between maternal vitamin D levels and adverse outcomes. Nutrients.

[20] Tahsin T, Khanam R, Chowdhury NH (2023). Vitamin D deficiency in pregnancy and the risk of preterm birth: a nested case-control study. BMC Pregnancy Childbirth.

[21] Wagner CL, Hollis BW (2018). The implications of vitamin D status during pregnancy on mother and her developing child. Front Endocrinol (Lausanne).

[22] Dunphy L, Tang AW (2023). Vitamin B(12) deficiency presenting with a pancytopenia in pregnancy. BMJ Case Rep.

[23] He J, Jiang D, Cui X, Ji C (2022). Vitamin B12 status and folic acid/vitamin B12 related to the risk of gestational diabetes mellitus in pregnancy: a systematic review and meta-analysis of observational studies. BMC Pregnancy Childbirth.

[24] Machamba AA, Azevedo FM, Fracalossi KO, do C C Franceschini S (2021). Effect of iodine supplementation in pregnancy on neurocognitive development on offspring in iodine deficiency areas: a systematic review. Arch Endocrinol Metab.

[25] Brantsæter AL, Garthus-Niegel S, Brandlistuen RE, Caspersen IH, Meltzer HM, Abel MH (2022). Mild-to-moderate iodine deficiency and symptoms of emotional distress and depression in pregnancy and six months postpartum - results from a large pregnancy cohort. J Affect Disord.

[26] Nykjaer C, Alwan NA, Greenwood DC, Simpson NA, Hay AW, White KL, Cade JE (2014). Maternal alcohol intake prior to and during pregnancy and risk of adverse birth outcomes: evidence from a British cohort. J Epidemiol Community Health.

[27] Marshall AT, Bodison SC, Uban KA (2022). The impact of prenatal alcohol and/or tobacco exposure on brain structure in a large sample of children from a South African birth cohort. Alcohol Clin Exp Res.

[28] Daggy JK, Silver RM, Guise D, Haas DM (2022). The impact of self-reported alcohol, tobacco, and recreational drug use during pregnancy on adverse pregnancy outcomes in first-time mothers [PREPRINT]. Am J Perinatol.

[29] Gold KJ, Marcus SM. (2008). Effect of maternal mental illness on pregnancy outcomes. Obstet Gynecol.

[30] Tomfohr-Madsen LM, Racine N, Giesbrecht GF, Lebel C, Madigan S (2021). Depression and anxiety in pregnancy during COVID-19: a rapid review and meta-analysis. Psychiatry Res.

[31] Biaggi A, Conroy S, Pawlby S, Pariante CM (2016). Identifying the women at risk of antenatal anxiety and depression: a systematic review. J Affect Disord.

[32] Alves AC, Cecatti JG, Souza RT (2021). Resilience and stress during pregnancy: a comprehensive multidimensional approach in maternal and perinatal health. ScientificWorldJournal.

